# An Experimental Evaluation of Indoor Localization in Autonomous Mobile Robots

**DOI:** 10.3390/s25072209

**Published:** 2025-03-31

**Authors:** Mina Khoshrangbaf, Vahid Khalilpour Akram, Moharram Challenger, Orhan Dagdeviren

**Affiliations:** 1 International Computer Institute, Ege University, 35100 Bornova, Izmir, Turkey; mina.khoshrangbaf@mail.ege.edu.tr; 2 Department of Computer Science, University of Antwerp and Flanders Make, Middelheimlaan, 2020 Antwerpen, Belgium; moharram.challenger@uantwerpen.be; 3 Department of Computer Engineering, Ege University, 35100 Bornova, Izmir, Turkey; orhan.dagdeviren@ege.edu.tr

**Keywords:** mobile robots, indoor tracking, localization, WiFi, Bluetooth, ultra wideband

## Abstract

High-precision indoor localization and tracking are essential requirements for the safe navigation and task execution of autonomous mobile robots. Despite the growing importance of mobile robots in various areas, achieving precise indoor localization remains challenging due to signal interference, multipath propagation, and complex indoor layouts. In this work, we present the first comprehensive study comparing the accuracy of Bluetooth low energy (BLE), WiFi, and ultra wideband (UWB) technologies for the indoor localization of mobile robots under various circumstances. In the performed experiments, the error margin of the WiFi-based systems reached 608.7 cm, which is not tolerable for most applications. As a commonly used technology in the existing tracking systems, the accuracy of BLE-based systems is at least 44.12% better than that of WiFi-based systems. The error margin of the BLE-based system in tracking static and mobile robots was 191.7 cm and 340.1 cm, respectively. The experiments showed that even with a limited number of UWB anchors, the system provides acceptable accuracy for tracking the mobile robots. Using only four UWB beacons in an environment of about 431 m^2^ area, the maximum error margin of detected positions by the UWB-based tracking system remained below 13.1 cm and 28.9 cm on average for the static and mobile robots, respectively. This error margin is 88.05% lower than that of the BLE-based system and 93.27% lower than that of the WiFi-based system on average. The high tracking precision, the need for a lower number of anchors, and the decreasing hardware costs point out that UWB will be the dominating technology in indoor tracking systems in the near future.

## 1. Introduction

Mobile robots, equipped with sensors and powerful computing capabilities, have applications in different areas. In healthcare, mobile robots are used for patient transportation, the delivery of medications and supplies, and the disinfection of rooms. In manufacturing and warehousing, mobile robots improve productivity and accuracy. They can transport materials and products within factories and warehouses, they can navigate dynamic environments using sensors, or they can conduct inventory checks, ensuring accurate stock levels and reducing the need for manual audits. In agriculture, mobile robots may perform various tasks such as plowing, seeding, and harvesting with high precision, reducing labor costs and increasing yield. They also may monitor crop health, detect diseases, and optimize irrigation and pesticide application. Mobile robots also have many applications in logistics and delivery, cargo handling, public safety and security, surveillance, and cleaning.

The spreading of autonomous mobile robots in different environments increases the demand for accurate tracking and localization systems. With the increasing demand for indoor positioning systems, researchers and companies have already started to study and develop different positioning and tracking systems. An ideal indoor tracking system should determine the exact location of the target robots in the environment. The GPS-based systems have very low accuracy in indoor environments; hence, in most applications, other technologies are used to track the mobile targets inside the buildings. The camera-based systems use image processing techniques to determine the location of the targets [1,2]. However, in these systems, the target should be visible by the cameras all the time. The obstacles or blind spots can reduce the accuracy of the camera-based systems. Radio frequency-based systems use different types of radio signals and methods to find the location of the target. These systems generally depend on the propagation speed, angle, and energy of the received radio signals [3]. However, the reflection of the signals and the effect of the obstacles on the energy level of the signals limits the accuracy of the radio frequency-based methods.

In this paper, we focus on the experimental evaluation of the radio frequency-based localization systems and perform a comprehensive and detailed analysis of the techniques and precision of the most common technologies. This paper focuses on the Bluetooth low energy (BLE), WiFi, and ultra wideband (UWB)-based localization systems. The main contributions of this paper are as follows:

- We provided a comprehensive evaluation of BLE, WiFi, and UWB-based localization systems for indoor environments, specifically focusing on their accuracy in tracking mobile robots for real-world scenarios.
- We found that WiFi-based robotic systems have significantly lower accuracy compared to BLE and UWB systems with error margins reaching 261.5 cm for static targets and 608.7 cm for mobile robots.
- BLE-based systems were found to be 44.12% more accurate than WiFi-based systems with error margins of 191.7 cm for static targets and 340.1 cm for mobile robots.
- From extensive evaluations, UWB-based systems demonstrated the highest accuracy with error margins of 13.1 cm for static targets and 29.9 cm for mobile robots. UWB systems were 88.05% more accurate than BLE and 93.27% more accurate than WiFi for tracking mobile targets.
- For mobile robot scenarios, we revealed the cost efficiency and ease of deployment of BLE beacons compared to UWB systems, which, despite their higher cost, offer significantly better accuracy.

The remaining parts of this paper are organized as follows: Section 2 provides brief information about the available radio frequency technologies and localization methods. Section 2.2 discusses the existing localization techniques using wireless signals. Section 4 presents the details of experiments, and Section 6 discusses the obtained results. Finally, Section 7 draws the conclusions.

## 2. Background

Most of the available indoor localization systems use radio waves and a localization method to find the coordinates of mobile targets. This section provides a brief survey about the radio signals and localization methods used in indoor localization systems.

### 2.1. Wireless Technologies

Radio waves are transmitted at speeds close to the speed of light and have different frequencies. Frequency is the number of vibrations of an electromagnetic wave per second measured in Hertz. Generally, Radio Frequency Identifier (RFID) [4], wireless sensor networks (WSNs) [5], WiFi [6], Bluetooth low energy (BLE) [7,8], and ultra wideband (UWB) [9,10] are the most common technologies which are widely used in the indoor positioning systems. Each technology has its own limitations, cost, and success ratio; hence, some hybrid systems [11,12] have been proposed to cover the limitations of each technology.

The Radio Frequency Identification Device (RFID) was originally developed for the storage and transmission of electromagnetic data, but later, it was used for the separation of entities and motion control [13]. An RFID system has at least one reader that can communicate with RFID tags. RFID systems are divided into active and passive systems. In active RFID systems, tags are powered by a power source (usually a small battery), periodically broadcasting their identity and some custom data. Passive RFID tags have no power source and have a short (usually less than 1 m) detection range. Most of the readers in RFID-based systems are expensive, and active tags may drain the battery very fast. Also, the limited communication range of passive tags significantly limits the use of this technology in positioning systems.

The IEEE 802.15.1 standard [14] which is also known as Bluetooth, uses short ultra-high-frequency radio waves between 2.4 and 2.485 GHz. The latest version of Bluetooth technology consumes lower energy and provides up to 100 m communication range. Various positioning systems have been developed based on Bluetooth signals [15,16]. The main idea is to estimate the distance between the sender and the receiver based on the Received Signal Strength Indicator (RSSI) value of the signal. However, relying only on RSSI values reduces the accuracy of BLE-based positioning systems because various factors such as reflection and obstacles may affect the RSSI value. In WiFi technology, an access point that usually operates at 2.4 GHz or 5 GHz frequencies provides a wireless gateway to the other devices. Generally, the access points have a communication range between 20 and 150 m. WiFi signals are almost everywhere; hence, WiFi-based indoor positioning systems have been the focus of numerous studies [17,18]. WiFi positioning systems use the access points in the environment as reference points to determine the location of the target entity. Ultra wideband (UWB) is a new technology that can be used for both communication and indoor localization. In UWB technology, the sender transmits short radio pulses of less than 1 nanosecond (ns) over 500 MHz or greater bandwidth with a frequency between 3.1 GHz and 10.6 GHz. Theoretically, the UWB-based positioning systems may reach up to 10 cm accuracy.

### 2.2. Localization Methods

Various algorithms and software methods are used to measure the distance based on the radio signals and determine the location of the target objects. In the trilateration [19] method, we measure the distance between the robot and at least three anchor nodes. Let i=1, 2, and 3 be the index of anchor nodes, and let $r_{i}$ be the measured distance from the mobile node to anchor *i*. By repeating the circle relation for each anchor as ${\left(x_{i}-x\right)}^{2}-{\left(y_{i}-y\right)}^{2}=r_{i}^{2}$, we will have three relations with two unknown variables *x* and *y*. The location of the mobile node will be the intersection of three circles.

The idea of the finger-printing method [20] is to create a map of received RSSI values for different locations of the environment. We may divide the environment into cells, record the RSSI value of the received signals in each cell, and create a map of RSSI values. The input of a finger-printing-based localization system is the RSSI value received by a mobile node, and the output is the coordinate of a matched location with the given RSSI value.

The Angle of Arrival (AoA) method [3] uses special antennas to find the angle of receiving signals. The position of the source node is calculated based on the angle, arrival time difference of the signals, and the distance between the receivers. In the AoA method, a line of sight must be maintained between the target and receiver nodes which is not feasible in most applications. Also, this method requires special and costly hardware and precise clock synchronization between the nodes.

In the Time of Arrival (ToA) or Time of Flight (ToF) method, the signal’s propagation time is used to find the distance between the sender and receiver [3]. Based on the d=v×t formula (*d* is the distance, *v* is the speed, and *t* is the time), the receiver can calculate the distance to the sender using the propagation time of the signal. To find the propagation time, the sender may attach the sending time to the message, but the sender’s and receiver’s clocks must be tightly synchronized, which is a difficult task. In another method, known as two-way ranging, the receiver immediately sends back the signal to the sender. So, the propagation time of the signal will be the half of the signal’s round trip time. The Time Difference of Arrival (TDoA) method uses the difference of sent and received times of signals instead of propagation time [3]. This method requires at least two receivers with tightly synchronized clocks. In this method, the receivers record the receiving time of the signals. The difference in arrival time at the two nodes gives the distance difference between the sender and the receivers based on the equation △d=v×△t. Using the value of △d and the coordinate of receivers, we can calculate the coordinate of the robot.

The weighted averaging method uses the RSSI values of the received signals and estimates the location of the target object by taking the weighted average coordinates of the surrounding anchor nodes [21]. The signals that come from closer anchor nodes will have higher RSSI values than the signals from far anchors. The robot uses the RSSI values as the weight of anchors and calculates its coordinates by taking the weighted averaging of the anchor’s positions. Many sophisticated variants of this method have been proposed in different studies; however, all of them depend on the RSSI values and the averages of the anchor’s coordinates.

## 3. Related Work

The accuracy of indoor localization systems has been the subject of many research studies from different perspectives. Most of the existing studies focus on improving the accuracy of localization systems [22,23,24], while some others discuss the advances and challenges of available technologies and techniques [3,25,26]. In this study, we evaluate the accuracy of existing technologies for the indoor localization of mobile robots. Hence, the studies that focus on improving the precision of localization systems is beyond this study.

Many surveys are available in the literature that discuss the techniques, drawbacks and expected accuracy of localization systems [26,27,28,29]. However, most of them are theoretical studies that only cover the technical specification of the approaches. The authors of [30] have presented a mathematical model to calculate the possible supported user density for multiple localization approaches using UWB indoor localization. To reduce the complexity of the UWB-based localization system, a position estimator for multiple anchors has been presented in [31]. The proposed method uses a Kalman filter and has been evaluated using mathematical models and a simulation environment. In [32], the effects of received signal strength on the path loss model parameters and the estimated distance in UWB-based systems have been evaluated in the simulation environment. A review on the self-calibration and collaborative localization systems using UWB nodes has been presented in [33]. The authors propose a classification and theoretical analysis to improve the performance of UWB systems.

On the other hand, the studies that evaluate the accuracy of the localization methods on the real world test beds generally focus on a specific technique or algorithms or perform limited experiments with restrictive assumptions. The authors of [34] have studied the accuracy of the Time Difference of Arrival method with UWB anchors and three localization algorithms. The performance of algorithms have been evaluated on a test bed with static and mobile targets. A specific UWB-based localization system for sport postures has been proposed in [35], where several UWB tags are attached to the human bodies and movements of different body parts are detected by the several anchor nodes, which are placed around the human body. The authors of [36] have proposed a hybrid approach that combine ultrasonic and bluetooth low energy localization systems to improve the accuracy of the system. The performance of the proposed approach has been evaluated on a test bed with 10 BLE beacons and 12 ultrasonic receivers.

An experimental comparison between RSSI-based and multi-carrier phase difference-based localization methods have been presented in [37]. In the experiments, random static points have been selected in a room for validation of the methods. Other hybrid localization methods based on the fusion of UWB and BLE [38], combining WiFi and UWB [39] or WiFi and BLE [40] have been proposed to achieve higher positioning accuracy and reduce the cost of the positioning system. Most of the proposed methods in this category has been designed for specific use cases or try to improve the accuracy under some important assumptions, which usually are not feasible in real-world applications.

The authors of [41] have reviewed the UWB, BLE, WiFi and Inertial Measurement Unit (IMU)-based localization systems. They have categorized these major approaches based on their technology, application area, and accuracy. The study is based on the theoretical data, and the approaches have not been tested in a real test bed. A UWB-based localization system has been proposed in [42] for smart factories. The proposed system uses Bayesian filtering to normalize the incoming data from the UWB and track the motions in the environment. The performance of the developed system has been evaluated by an experimental test in the laboratory facility.

A notable advancement in UWB-based indoor localization systems is the implementation of the Downlink Time Difference of Arrival (DL-TDoA) feature [43], which allows UWB-enabled mobile devices to determine their location by passively receiving signals from synchronized anchor infrastructures. This method enhances user privacy, scalability, and energy efficiency, making it ideal for complex environments like airports and shopping malls. Additionally, integrating deep learning techniques, such as Long Short-Term Memory (LSTM) networks, with UWB systems has improved localization accuracy. By training on distance measurements from anchors, these models can predict user positions with minimal errors, addressing challenges like non-line-of-sight conditions and computational complexities associated with traditional algorithms [44]

Similarly, BLE has evolved with the integration of multi-modal data fusion and machine learning approaches to enhance indoor positioning accuracy. Combining BLE signals with other data such as visible light positioning (VLP) has been explored to overcome individual limitations. For instance, a system that fuses BLE and VLP data using deep neural networks achieved a mean localization error of 20 cm, significantly outperforming individual technologies [45]. Moreover, researchers have been leveraging deep learning models to process BLE signal characteristics, including Received Signal Strength Indicator (RSSI) values and AoA estimations [46]. These advancements contribute to more accurate and robust indoor localization solutions. To the best of our knowledge, most of the existing studies focus on a specific technology or present theoretical comparisons. In this work, we provide a comparative evaluation between the accuracy of different localization systems using experimental evaluations and real mobile robots.

## 4. Accuracy Evaluation

**Figure 1:**
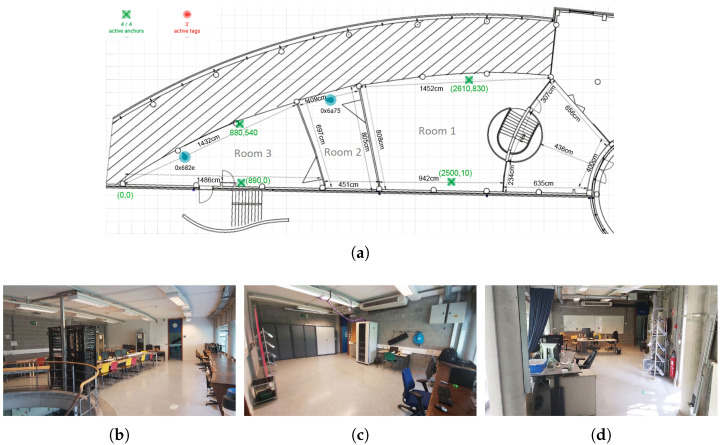
(**a**) The floor plan, (**b**) room 1, (**c**) room 2, and (**d**) room 3 of the experimental environment.

To evaluate the accuracy of different wireless technologies, we implemented three localization systems using BLE, WiFi, and UWB signals and measured the difference between estimated and real coordinates of mobile and static robots in different locations of an indoor environment with 431 m^2^ area. Figure 1a shows the floor plan of the environment with three rooms that we used for experiments. In this figure, the numbers next to each wall show the length of the wall in centimeters. The green cross symbols show the UWB anchors and the blue symbols show the mobile robots. The numbers next to each anchor show the coordinates of the anchor. The (0,0) coordinate is the left-bottom corner of the map. Figure 1b–d show the three rooms in the experimental environment.

The number of anchor nodes used for each technology was selected based on standard deployment recommendations and practical considerations. Generally, UWB technology requires fewer anchors due to its ToF measurement capability, which provides more accurate distance estimations than the RSSI-based methods. The selected four-anchor configuration follows common deployment practices based on the range of tag and beacon anodes and was sufficient to cover the entire test area with high accuracy. BLE and WiFi rely on RSSI-based localization, which is more sensitive to environmental factors such as signal attenuation and multipath effects. To improve accuracy, we deployed a denser network of nine anchors to ensure that each point in the test areas is covered by at least three beacon nodes. While adding more UWB anchors may further refine accuracy, the existing literature suggests diminishing returns beyond a certain point. For instance, a study on UWB-based indoor localization observed that increasing the number of anchors beyond four in a limited area did not significantly enhance localization accuracy [47].

The placement of all anchors was determined based on best practices for each technology. We positioned anchors to ensure maximum coverage and minimal interference while maintaining line of sight where possible, particularly for UWB technology. For BLE/WiFi, anchor placement was optimized empirically by measuring signal strength distributions across the environment and ensuring redundancy in coverage. In BLE/WiFi anchor placement, we tried to cover all positions in the environment with at least three anchor nodes. Table 1 shows the coordinate of the placed UWB and BLE/WiFi anchor nodes.

The experimental layout was carefully selected to represent a realistic indoor environment with varying signal propagation conditions. The presence of multiple rooms, walls, and open spaces allowed to assess the impact of obstructions and interference on each localization technology. However, different layouts may lead to variations in accuracy. In the environments with increased obstructions, BLE and WiFi systems may experience greater degradation due to multipath effects and signal attenuation, whereas the ToF-based approach is more resilient to such challenges. Additionally, larger areas may require a higher number of anchors to maintain accuracy across all technologies.

In the static mode, we placed three static robots in different rooms and recorded the detected coordinates by the localization system. In the mobile mode, the robot moved from the most right coordinate of the first room to the most left location in the third room. During the movement, we recorded the estimated location by different localization systems.

### 4.1. Setup for BLE and WiFi Experiments

**Figure 2:**
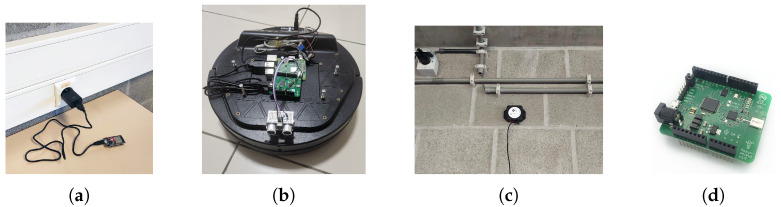
(**a**) NodeMCU ESP32, (**b**) Kobuk Robot equipped with Raspberry Pi and UWB tag device, (**c**) UWB anchor node, and (**d**) UWB tag node.

In the BLE and WiFi-based systems, we used nine NodeMCU-ESP32 devices (produced by AI-Thinker Co., Ltd., Shenzhen, China) to probe the broadcasted BLE or WiFi signals from the mobile target. The NodeMCU-ESP32 device has both WiFi and BLE modules and an Xtensa 32-bit LX6 microprocessor. Figure 2a shows a NodeMCU device that we used in BLE and WiFi-based experiments. For the mobile target, we used a Kobuki robot (developed by Yujin Robot Co., Ltd., Incheon, Republic of Korea) equipped with a Raspberry Pi 3B (Figure 2b). The robot broadcasted BLE signals using Raspberry Pi with 3 Hz frequency and −56 dBm transmission power. For the WiFi-based experiments, we started a WiFi hot spot application in the Raspberry Pi, so the device broadcasted its SSID over WiFi signals. The BLE and WiFi signals were received by the NodeMCU devices in the environment, and their RSSI was measured by the receivers.

**Figure 3:**
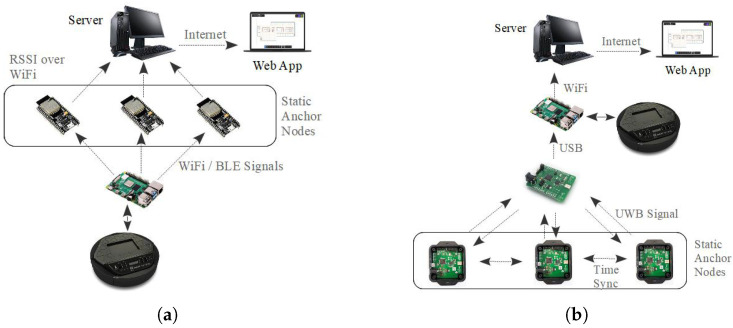
The general architecture of (**a**) WiFi and BLE, and (**b**) UWB-based localization systems.

We implemented an embedded program to run on the ESP nodes and a NodeJS-based web application to collect the information and monitor the real-time location of the anchor and mobile nodes. The embedded program in the ESP nodes measures the strength of the received BLE and WiFi signals by the ESP device and sends the detected signal strength and the signal source ID to a web server over HTTP protocol. The server collects the received data from all ESP nodes and determines the location of the mobile node using the weighted averaging method. Figure 3a shows the general architecture of WiFi and BLE-based localization systems.

### 4.2. Setup for UWB Experiments

For the UWB-based experiments, we used PoZYX UWB (PoZYX Co., Ltd., Ghent, Belgium) devices. The PoZYX UWB tracking system consists of anchor and tag devices. The anchor devices (Figure 2c) are placed in the environment and broadcast UWB signals. The tag device (Figure 2d) is attached to the mobile object and uses the received signals from the anchor nodes to determine its location. The PoZYX UWB-based tracking system uses the two-way-ranging (TWR) technique to calculate the coordinates of the target robot. In TWR, the distance between a mobile tag and an anchor node is calculated by sending a UWB signal and receiving it back from the anchor. The distance is calculated by measuring the time of sent and received signals. To find the position of the mobile tag, the tag initiates communication with the anchors one by one and finds the distance to them. If the mobile tag can calculate its distance with at least three anchors, it can estimate its position using the trilateration method. The collected position data are forwarded to the Raspberry Pi by the tag over the USB port. The program running on Raspberry Pi forwards the incoming location data to a web server. Figure 3b shows the general architecture of the UWB-based localization system.

## 5. Results

**Figure 4:**
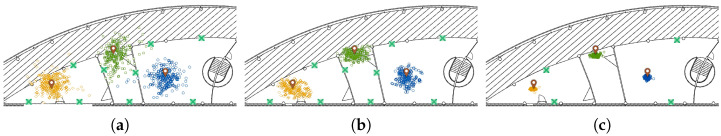
Estimated locations for the static robots using (**a**) WiFi, (**b**) BLE, and (**c**) UWB systems.

Figure 4 shows the estimated locations for the static robots using WiFi, BLE, and UWB systems. In this figure, the brown symbols show the correct location of robots and the points show the estimated location of nodes. In the WiFi-based system (Figure 4a), the estimated locations for the static robots can be far from the correct location. The distribution of points in Figure 4a shows that the WiFi-based localization system reports locations that can be very far from the correct location. This figure also shows that the WiFi-based system can locate the robots in the incorrect room. Figure 4b shows the reported locations for the static node by the BLE-based system. The figure clearly shows that the accuracy of the detected location is better than that of the WiFi-based system; however, most of the detected locations are still far from the correct location. The BLE system always locates the target static nodes in the correct room, but most of the detected coordinates have a considerable error margin. Figure 4c shows the detected locations for the static nodes in the UWB-based system. The figure shows that most of the reported locations are very close to the correct target locations. In contrast to the BLE and WiFi-based systems, the detected locations by the UWB-based system have very limited error margins.

**Figure 5:**
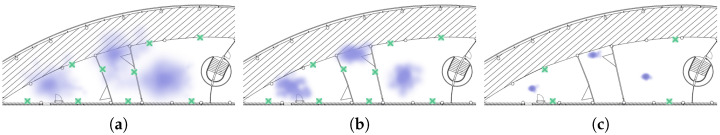
Heat map of the estimated positions for the static robot using (**a**) WiFi, (**b**) BLE, and (**c**) UWB systems.

Figure 5 shows the heat map of the detected locations for the static robots by different localization systems. Figure 5a shows that the WiFi-based localization system may report incorrect locations for the robot in a big area of the environment. Despite the fact that the robots are static, the WiFi-based system reports many different locations in the entire environment. Figure 5b shows that the BLE-based system has considerably better accuracy than the WiFi-based system. The error range of the reported locations in the BLE-based system is limited to the inside of the rooms, while in the WiFi-based system, the detected locations may fall in the incorrect room. Figure 5c shows that the UWB-based system finds more correct locations of the robots with a limited error range.

**Figure 6:**
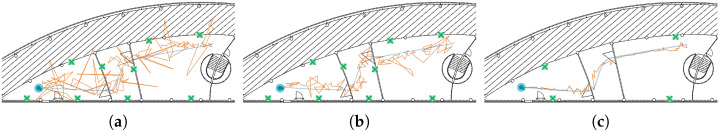
Estimated paths for the mobile robot using (**a**) WiFi, (**b**) BLE, and (**c**) UWB.

Figure 6 shows the correct (blue line) and estimated path (orange line) for the mobile robot using WiFi (Figure 6a), BLE (Figure 6b), and UWB (Figure 6c) systems. Figure 6a shows that in the WiFi-based localization system, the distance between estimated and correct samples on the path is relatively high. Figure 6b shows that the distance between the sample points of the estimated and correct path in the BLE-based system is lower than that of the WiFi-based system. Despite the relatively high number of anchor nodes (nine ESP nodes), the accuracy of the WiFi and BLE-based system is much lower than that of the UWB-based system. Figure 6c shows that the UWB-based system has estimated a close path to the correct traversed path with only four anchor nodes.

**Figure 7:**
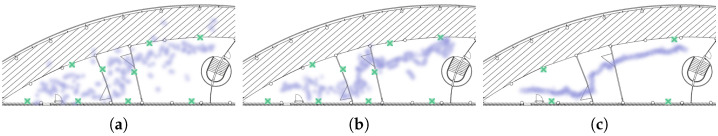
Heat map of the estimated paths for the mobile robots using (**a**) WiFi, (**b**) BLE, and (**c**) UWB.

Figure 7 shows the heat map of estimated paths for the robot in WiFi (Figure 7a), BLE (Figure 7b), and UWB (Figure 7c) systems. This figure shows that the error margin of UWB-based localization is much lower than that of the BLE and WiFi-based systems. Although the BLE and WiFi-based systems have five more anchor nodes than the UWB-based system, their detected path has a higher error ratio.

## 6. Discussion and Comparison

**Figure 8:**
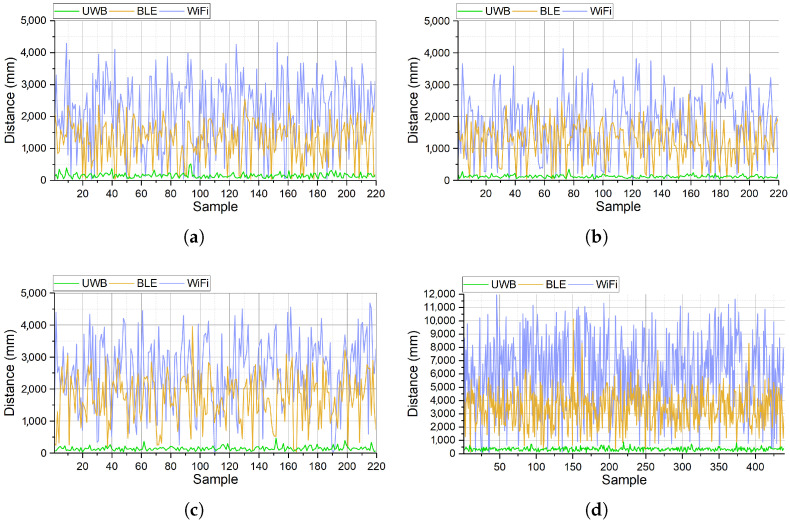
Distance between the estimated and correct location of the (**a**) first static, (**b**) second static, (**c**) third static, and (**d**) mobile robot.

This section provides a comparison of the accuracy of the evaluated systems. Figure 8 shows the distance between the detected and correct location of robots in static and mobile modes. In this figure, the blue lines show the distances of detected locations in the WiFi-based system, the orange lines show the distances of detected locations in the BLE-based system, and the green lines show the distances of detected locations in the UWB-based system. Figure 8a shows the distance between the detected and correct location of the static robot in room 1. The figure shows that the error margin of the UWB-based system is less than 500 mm in all samples, while this margin reaches 2500 mm for BLE and 4200 mm in the WiFi-based system. Similarly, Figure 8b and Figure 8c, respectively, show the distance between detected and correct locations of static robots in rooms 2 and 3. The error margin of detected locations using the UWB-based system is less than 420 mm and 250 mm in rooms 2 and 3, respectively. The maximum error margin of the BLE-based system in rooms 2 and 3 is more than 3900 mm, while this value for the WiFi-based system is more than 4600 mm (room 3). Figure 8 indicates that for the static targets, the error margin of the UWB-based system is less than 0.5 m, which is 89.13% lower than the WiFi and 87.17% lower than the BLE-based system. For the mobile target, the maximum error margin of the UWB-based system is 920 mm, while the error margin for the BLE and WiFi-based systems is 10,050 mm and 12,000 mm, respectively. This indicates that in the worst case, for the mobile targets, the UWB-based system is at least 90.8% more accurate than the BLE system and 92.33% more accurate than the WiFi-based system.

**Figure 9:**
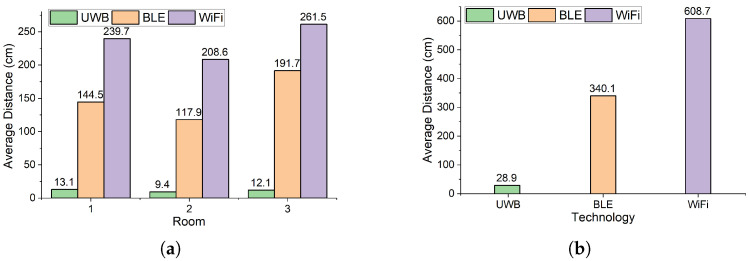
(**a**) Average distance between detected and correct locations of static robots. (**b**) Average distance between detected and correct locations of target mobile nodes.

Figure 9 shows the average distance of all sample points between the detected and correct location of static (Figure 9a) and mobile robots (Figure 9b). The error distance for each recorded position is computed as the Euclidean distance between the estimated and actual positions. The average error is obtained by summing all individual errors and dividing by the total number of recorded positions:

$$\mathrm{Average}\;\mathrm{Error}=\frac{1}{N}\sum_{i=1}^{N}\sqrt{{\left(x_{i}-x_{\mathrm{true}}\right)}^{2}+{\left(y_{i}-y_{\mathrm{true}}\right)}^{2}}$$

where $\left(x_{\mathrm{true}},y_{\mathrm{true}}\right)$ represents the correct position of robots. For the static robots (Figure 9a), the average error distance for the UWB-based system is less than 13.1 cm in all rooms, while these values for the BLE and WiFi-based systems are 191.7 cm and 261.5 cm, respectively. Figure 9a indicates that the average error margin of the UWB-based system is 93.2% and 95% lower than that of the BLE and WiFi systems, respectively. The average error margin of the BLE-based system for the static targets is at least 36.41% lower than that of the WiFi-based systems. For the mobile robots (Figure 9b), the average error distance for the UWB-based system is 28.9 cm, while this value for the BLE and WiFi-based systems is 340.1 cm and 608.7 cm, respectively. Therefore, the average error margin of the UWB-based system for the mobile targets is 91.5% and 95.25% lower than the BLE and WiFi-based systems. The average error margin of the BLE-based system for the mobile targets is 44.12% lower than that of the WiFi-based system.

**Figure 10:**
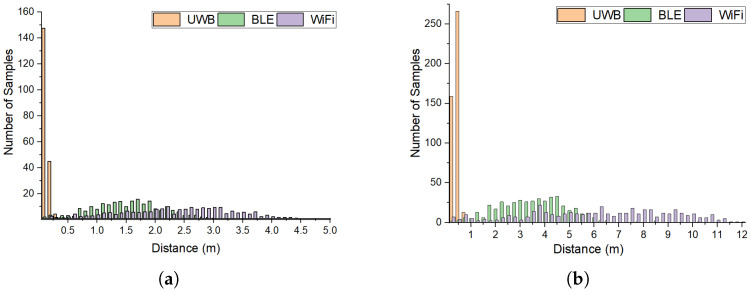
The histogram of reported locations by the WiFi, BLE and UWB-based localization system for (**a**) static and (**b**) mobile robots.

Figure 10 shows the error histogram of reported locations by the WiFi, BLE, and UWB-based localization systems for static (Figure 10a) and mobile robots (Figure 10b). For the static robots, we took the average of errors for all three rooms. Figure 10a shows that in the UWB-based system, the average error margin of all reported locations for the static robots is less than 0.3 m, while this value for the BLE and WiFi-based systems is 2.9 m and 4.5 m, respectively. This indicates that for the static robots, the average error ratio of the UWB-based system is 89.65% less than that of the BLE system and 93.3% less than that of the WiFi-based system. For the mobile targets (Figure 10b), the error ratio of the UWB-based system is less than 0.8 m, while this value for the BLE and WiFi-based systems is 6.7 m and 11.9 m, respectively. This indicates that for the mobile robots, the error ratio of the UWB-based system is 88.05% less than the BLE system and 93.27% less than the WiFi-based system.

**Figure 11:**
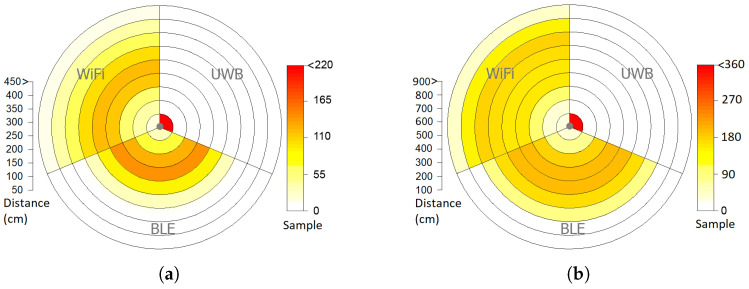
The accuracy of the WiFi, BLE and UWB-based localization systems for (**a**) static and (**b**) mobile robots.

Figure 11 shows the accuracy of WiFi, BLE and UWB-based localization systems for static (Figure 11a) and mobile (Figure 11b) robots. Figure 11a,b show that all reported samples by the UWB-based system have less than 50 cm distance to the correct location of the static target and less than 100 cm distance to the correct location of the mobile target. For the BLE-based system, the distribution of distances between reported and correct positions of the static targets is in the range of 150 cm and 250 cm, while this range for the mobile targets is in the range of 200 and 600 cm. For the WiFi-based system, the majority of distances are in the range of 100 cm and 400 cm, while this range for the mobile targets is between 300 and 700 cm.

## 7. Conclusions

While UWB inherently offers higher precision due to its time-of-flight measurement capabilities, recent enhancements in BLE, particularly through multi-modal fusion and deep learning, have narrowed the performance gap. Recent studies have explored hybrid UWB-BLE approaches, machine learning-based localization, and improved filtering techniques such as Kalman filters for enhanced accuracy. BLE has also seen improvements in localization through AoA methods and hybrid BLE-UWB approaches.

In this research, we evaluated the accuracy of common localization technologies to locate the mobile robots in the indoor environment. We implemented three different localization systems using BLE, WiFi, and UWB technologies. Despite the lower cost and almost straightforward establishment process, our experiments showed that the accuracy of WiFi-based systems is lower than that of BLE and UWB-based systems. Based on our experiments, the error margin of the WiFi-based systems for tracking the static and mobile robots can reach 261.5 cm and 608.7 cm, which is not acceptable for most applications.

BLE is the most common technology that is used in the current tracking systems. The BLE beacons are cost-efficient and can be easily deployed in the target environment. Our experiments showed that the BLE-based system is 44.12% more accurate than the WiFi-based systems. The error margin of the BLE-based system in the tracking of static and mobile targets is 191.7 cm and 340.1 cm, which can be tolerated by some applications that track the targets in relatively large environments such as shopping centers or hospitals.

UWB is a new and most promising technology for indoor tracking systems. Our experiments showed that even with a limited number of UWB beacons, the accuracy of the system is acceptable in most applications. Using only four UWB beacons, the maximum error margin of the implemented tracking system remained under 13.1 cm and 29.9 cm for the static and mobile targets, respectively. The experiments showed that on average, the UWB-based system is 88.05% more accurate than the BLE system and 93.27% more accurate than the WiFi-based system for tracking the mobile targets. Despite the dominant accuracy, the cost of UWB-based tracking systems is higher than that of the BLE and WiFi-based systems.

Generally, UWB remains a promising technology across different autonomous robots and layouts due to the following:

- Scalability: UWB is independent of the specific mobile robot as long as the robot is equipped with a UWB tag.
- Robustness: Unlike BLE and WiFi, UWB is less affected by signal interference from moving objects or environmental changes.
- Flexibility in Deploymen: While layouts with more obstacles may require additional anchors, UWB’s ToF-based method ensures consistent accuracy in diverse settings.

While the fundamental performance trends observed in this study are expected to remain consistent, site-specific factors such as anchor placement feasibility, interference sources, and the material composition of walls can influence absolute error values. Future studies could extend our findings by testing these localization systems in a variety of indoor layouts.

As the future works, the performance of different localization techniques can be measured using UWB signals for both static and mobile robots. Also, the accuracy of UWB and BLE-based systems can be evaluated with different numbers of anchor nodes. To measure the accuracy of a UWB-based system with additional anchor nodes, different numbers of anchors can be added to the same environment, and multiple experiments can be conducted with varying numbers of anchors.

Furthermore, a hypothesis test can be applied to assess whether UWB’s superior accuracy is statistically significant. For instance, a one-way ANOVA or a *t*-test could be used to compare the error margins across different technologies. The null hypothesis ($H_{0}$) would state that there is no significant difference in localization accuracy among UWB, BLE, and WiFi, while the alternative hypothesis ($H_{1}$) would state that UWB provides significantly higher accuracy.

## Figures and Tables

**Table 1:** Coordinates of the placed anchor in the environment.

UWB	Anchor	Room	BLE/WiFi	Anchor	Room
1	(890, 0)	3	1	(290, 0)	3
2	(880, 540)	3	2	(620, 0)	3
3	(2500, 10)	1	3	(895, 560)	3
4	(2610, 830)	1	4	(1470, 450)	2
			5	(1710, 0)	2
			6	(1920, 390)	2
			7	(2050, 795)	1
			8	(2600, 0)	1
			9	(2650, 835)	1

## Data Availability

Data are contained within the article.
